# Spatial transcription of CYP1A in fish liver

**DOI:** 10.1186/1472-6793-7-12

**Published:** 2007-12-05

**Authors:** Pål A Olsvik, Kai K Lie, Øystein Sæle, Monica Sanden

**Affiliations:** 1 National Institute of Nutrition and Seafood Research, N-5817 Bergen, Norway; 2 Department of Biology, University of Bergen, N-5020 Bergen, Norway

## Abstract

**Background:**

The aim of this work was to study how evenly detoxifying genes are transcribed spatially in liver tissue of fish. Ten Atlantic salmon *Salmo salar *were intraperitoneally injected with 50 mg/kg of the strong CYP1A inducer β-naphthoflavone and liver tissue harvested seven days later. The liver from 10 control and 10 exposed fish were split into eight sections, RNA extracted and three reference (β-actin, elongation factor 1A_B _(EF1A_B_)) and two detoxifying genes (CYP1A and GST) quantified with real-time RT-PCR. The cellular localization of the EF1A_B _and CYP1A mRNA in the liver of control and β-naphthoflavone treated fish was then determined by *in situ *hybridization (ISH) using EF1A_B _and CYP1A biotinylated oligonucleotide probes.

**Results:**

The study shows that genes encoding phase I and phase II conjugating enzymes are unevenly transcribed in different parts of the liver of Atlantic salmon seven days after a single-dose of β-naphthoflavone exposure. Transcription of CYP1A and GST was higher in the middle section of the liver compared to the distal and proximal parts of the organ. The ISH data suggest that CYP1A transcription happens mainly in hepatocyte cells in the liver, and that hepatocytes in the vicinity of blood vessels respond stronger to β-naphthoflavone than cells further away from the blood supply.

**Conclusion:**

Overall, the qRT-PCR and ISH results reported here suggest that gene expression analysis should be performed on as pure cell populations as possible. If bulk tissue samples are to be used, one should always check how evenly the target genes are expressed in tissue sections and organs in every study.

## Background

The liver is the largest internal organ and one of the most studied in fish, making up about 1% of total body mass in Atlantic salmon *Salmo salar*. It plays a central role in metabolism of nutrients absorbed in the digestive tract but also in metabolism and detoxification of many toxicants accompanying the foodstuff. The liver receives blood via the vena portae hepatica (70–80%) and the arteria hepatica. Nutrients and toxicants absorbed in the digestive track spreads throughout the liver from the vena portae hepatica on the distal part of the organ. The liver filters blood through a network of sinusoids formed by cuboidal hepatocytes. In fish, the liver does not contain discrete lobules bordered by septa, portal veins and bile ducts [1]. Eventually, the blood leaves the liver via the vena hepatica. Fish liver consists of several cell types; hepatocytes, which may represent up to 90% of total liver mass, fat storing stellate cells, phagocytic Kupffer cells, endothelial cells forming the fenestrated lining of the sinusoids and bile duct epithelial cells [2,3].

In most gene expression studies, a piece of the liver is sliced off, and RNA extracted from this particular part of the organ. It is considered to be of crucial importance to cut off the same section of the liver to ensure that one is examining exactly the same piece of tissue from fish to fish. Gene expression profiling or single-gene qPCR analysis is then performed on RNA extracted from this particular part of the liver. In order to check how evenly stress-responsive genes are expressed spatially and between different cell types in Atlantic salmon liver, two of the most studied detoxifying genes, CYP1A and glutathione S-transferase (GST) were selected, and the transcription levels measured throughout the liver. To extend these studies, *in situ *mRNA hybridization was used to examine if CYP1A and the reference gene elongation factor 1α are evenly expressed in different cell types but also spatially within the same cell types. The strong cytochrome P450 CYP1A inducer β-naphthoflavone (BNF) was used to increase the transcription of these genes in fish tissues.

*In situ *hybridization (ISH) is a useful technique for determining spatial patterns of gene expression within a particular tissue. ISH was introduced in 1969 [4,5] and allows for the cytological localization and visualization of specific transcripts at a single cell level. Our newly developed ISH protocol uses short biotin-labeled oligonucleotide probes (48 bp) and has been used with success to locate dietary and naked DNA in formalin-fixed, paraffin embedded intestinal tissue of Atlantic salmon [6]. Oligonucleotide probes generated with an automated DNA synthesizer penetrate cells more readily compared to longer probes (e.g. cRNA probes), are very stable and produce excellent hybridization signals [7].

**Figure 1:**
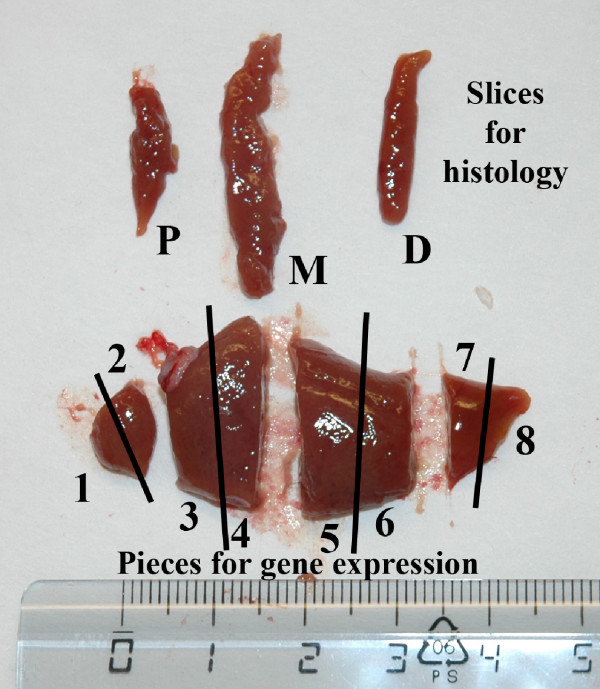
Atlantic salmon *Salmo salar *liver partitioned into eight transversal sections for transcriptional analysis and three transversal cross-sections for *in situ *hybridization. P = Proximal, M = Mid, D = Distal. Scale bar = cm.

In this study the goal was to examine the macroscopic distribution and cellular localization of two detoxifying genes and of three reference genes to evaluate if these are evenly expressed throughout the different parts of the Atlantic salmon liver. For this reason, the liver was cut transversally into eight parts (Fig. 1), and RNA extracted from each part for quantitative qRT-PCR analysis in control fish as well as in fish exposed to the strong CYP1A inducer BNF. We also sliced off three cross-sections of the liver for histological examination, to evaluate if two of the studied genes are evenly expressed in different cell types. The three sections were cut transversally from the proximal, mid and distal regions of the liver. *In situ *hybridization was used to examine the spatial patterns of gene expression of one detoxifying gene (CYP1A) and one reference gene (elongation factor 1A_B _in anatomically different areas of the liver.

## Results

**Figure 2:**
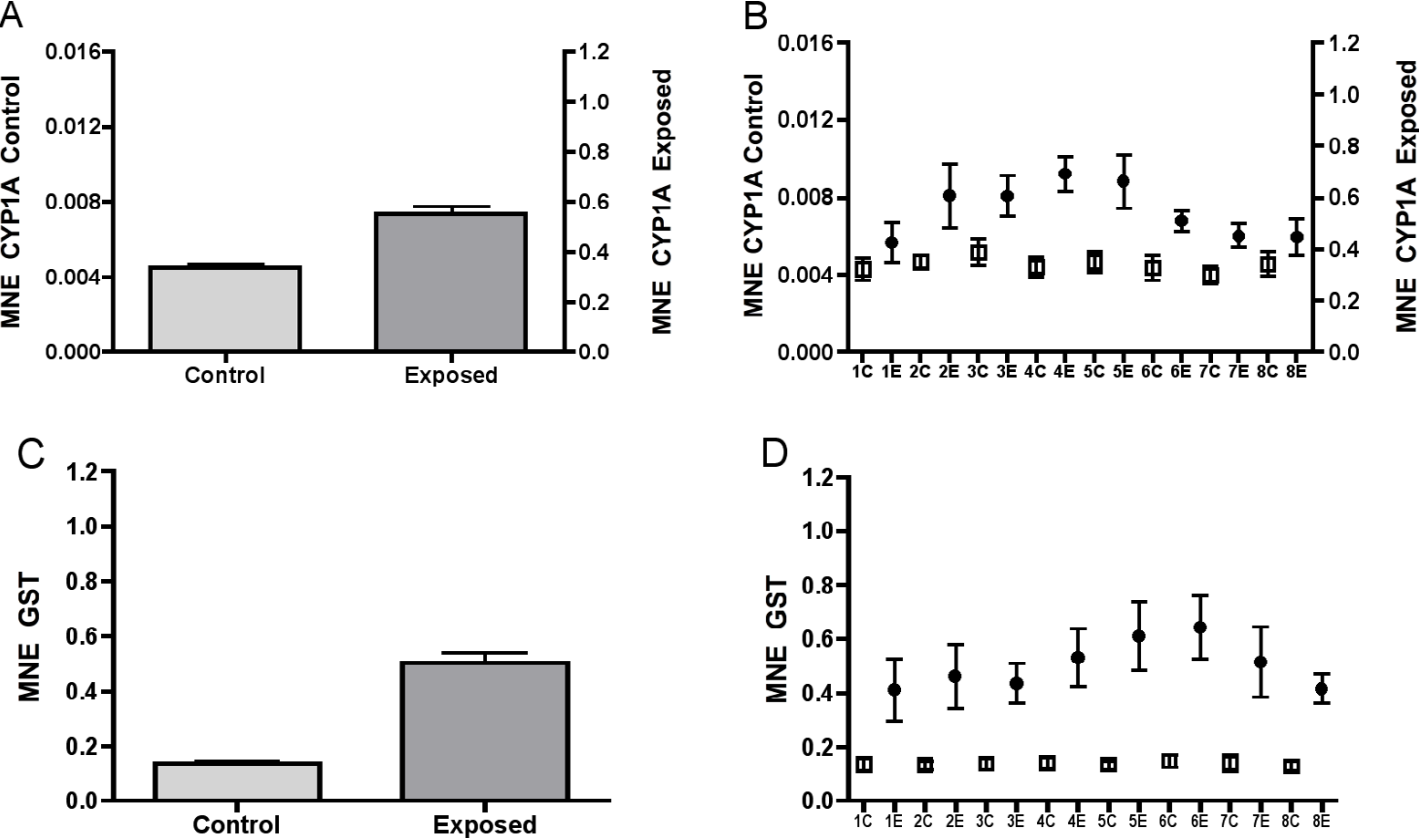
Mean normalized expression (MNE) of CYP1A (A) and GST (C) in eight sections (1–8) of Atlantic salmon *Salmo salar *liver from fish exposed to β-naphthoflavone. n = 6. Average MNE of CYP1A is shown in (B) and of GST in (D). n = 48. ● = exposed, □ = control. Note different axis for CYP1A in control and exposed fish.

Fig. 2 shows the induction of CYP1A and GST mRNA in liver of Atlantic salmon seven days after BNF-exposure. When data from eight liver sections from each of six control and six exposed fish are compared, the results show that CYP1A mRNA was 121 fold higher expressed in the exposed animals (Fig. 2A) (t-test, P < 0.0001). In comparison, GST mRNA was only 3.6 times higher expressed in exposed animals than in control fish (Fig. 2C) (t-test, P < 0.0001). In these analyses each of the eight sections from the same fish were treated as individual measurements. Pooling the eight measurements from each fish (n = 6 in each group), and recalculating the data with a non-parametric test yields approximately the same result. Spatial distribution of CYP1A in the eight liver sections is shown in Fig. 2B. In non-exposed animals, CYP1A mRNA expression was evenly expressed throughout the liver, and no significant differences were observed between the sections. The results show that baseline levels of CYP1A mRNA are relatively low in un-stressed Atlantic salmon liver. In contrast, expression patterns in exposed fish show that CYP1A mRNA levels are higher in the middle sections of the liver compared to the proximal and distal regions. The differences in CYP1A mRNA expression between the eight sections of liver were significant (Kruskal-Wallis, P = 0.0001). For GST mRNA, the same general expression pattern was found (Fig. 2D). In control fish, GST was evenly expressed in all eight sections, with very low standard deviations within the groups. In stressed fish, however, GST mRNA expression was highest in the middle part and towards the distal part of the liver, with highest levels in section five and six. The mRNA expression differed significantly between the sections (Kruskal-Walls ANOVA, P < 0.0001). Posthoc tests showed that the biggest difference was between section six and section eight (Dunn's multiple comparison test).

**Figure 3:**
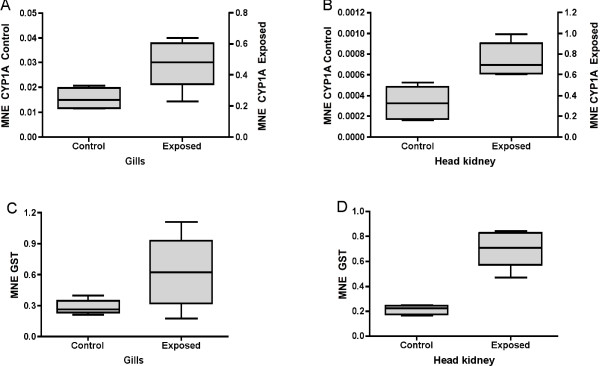
Mean normalized expression (MNE) of CYP1A and GST in gills (A and C) and head kidney (B and D) of Atlantic salmon *Salmo salar *exposed to β-naphthoflavone.

In order to check how the BNF treatment affected other organs in the fish, mRNA expression levels of CYP1A and GST were also quantified in gills and head kidney (Fig. 3). Measurements were obtained from the same six individuals that were used to study expression patters in liver. The results revealed that BNF acted as a strong CYP1A inducer also in these organs. In gills (Fig. 3A), CYP1A mRNA expression showed a 31-fold induction (t-test, P < 0.0001). Baseline CYP1A mRNA levels in head kidney were very low (Fig. 3B). The transcription level of this gene was 2261 fold higher in BNF-exposed animals compared to control animals (t-test, P < 0.0001). GST mRNA induction in exposed animals was more modest in gills and head kidney. The fold change for GST mRNA expression was 2.2 in gills and 3.3 in head kidney; the expression differences in the latter organ were significant (t-test, P < 0.022).

**Figure 4:**
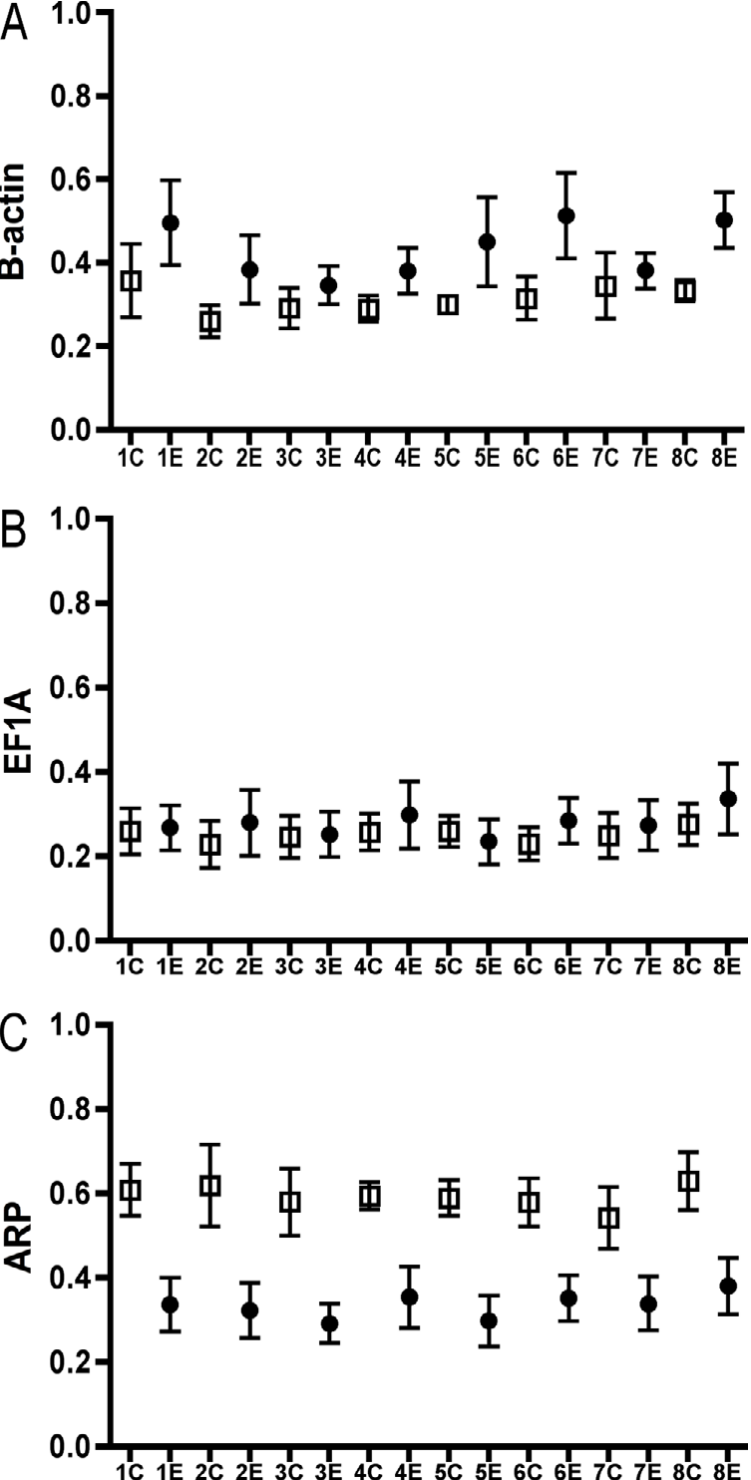
Reference gene stability in eight sections of Atlantic salmon *Salmo salar *liver. Y-axis = quantities transformed from raw Ct values. ● = exposed, □ = control.

In this work, the transcription levels of three reference genes were quantified, and a normalization factor calculated by the *geNorm *software used to quantify mean normalized expression of CYP1A and GST. In liver tissue, β-actin was the most stable reference gene, whereas EF1A_B _and ARP were the most stable reference genes in gills and head kidney, respectively. These stability measurements were based on the *M *value, as calculated by the *geNorm *software. Figure 4 shows the stability of each the three reference genes as quantities across the eight sections of liver. The highest relative quantities for each gene are set to 1. These raw, not yet normalized, reference gene quantities are the required data input for geNorm. The quantities are not saying anything about up- or downregulation *per se*. They can, however, be used to visualize reference gene stability. Based on the quantities, these results indicate that EF1A_B _(Fig. 4B) was stable within both the control and exposed groups, and that ARP (Fig. 4C) seems to be downregulated in the exposed group as compared to the control group. But since the stability of ARP was uniform within the control and exposed groups (Kruskal-Wallis, Control P = 0.99, Exposed P = 0.98), this gene can still be used in calculations of the normalization factor in examinations of spatial target gene expression throughout the liver.

**Figure 5:**
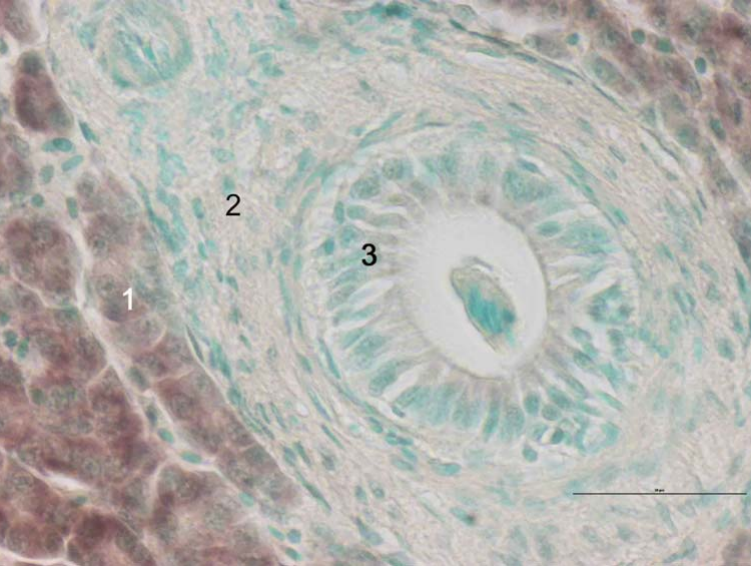
*In situ *hybridization staining of CYP1A (brown) in liver of Atlantic salmon *Salmo salar *exposed to β-naphthoflavone. Nucleus staining with methyl green. 1: hepatocytes, 2: connective tissue, 3: bile duct. Scale bar = 50 μm.

**Figure 6:**
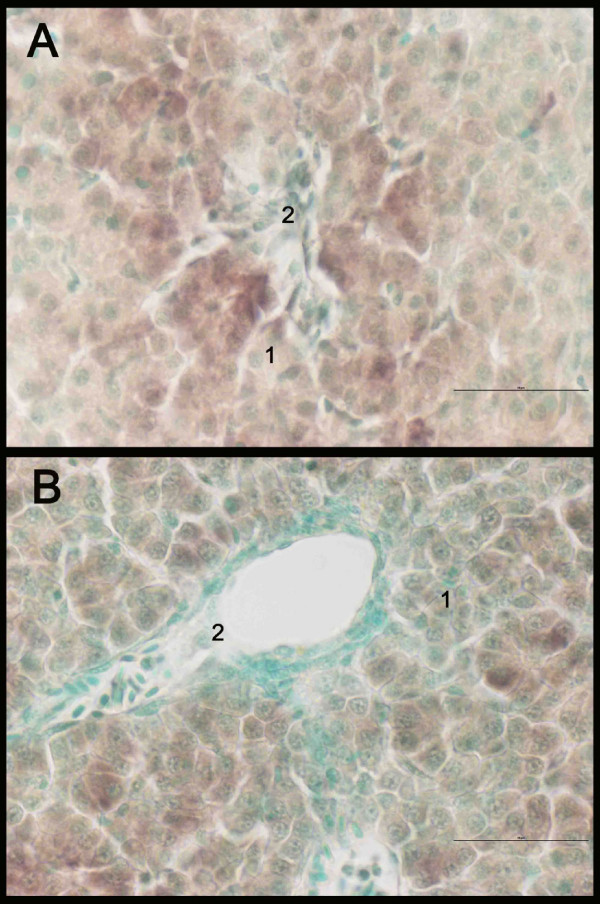
*In situ *hybridization staining of CYP1A (brown) in liver of Atlantic salmon *Salmo salar *exposed to β-naphthoflavone (A) and control (B). Nucleus staining with methyl green. 1: hepatocytes, 2: blood vessel. Scale bar = 50 μm.

**Figure 7:**
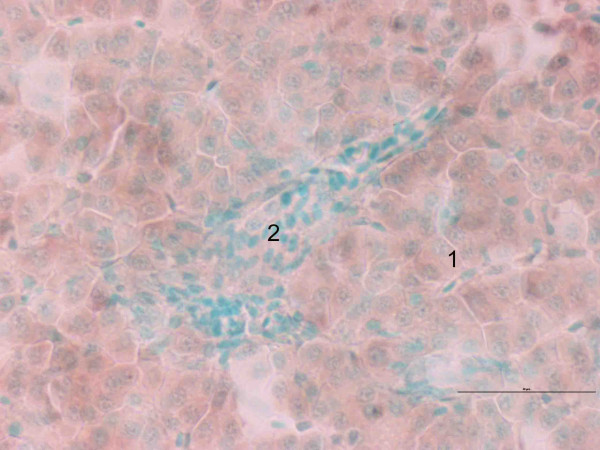
*In situ *hybridization staining of EF1A_B _(brown) in liver of Atlantic salmon *Salmo salar *exposed to β-naphthoflavone. Nucleus staining with methyl green. 1: hepatocytes, 2: blood vessel. Scale bar = 50 μm.

Expression patterns of CYP1A were the same in the different liver sections (data not shown). CYP1A was only expressed in hepatocytes, in both groups (Fig. 5). In Fig. 5 it can be clearly seen that CYP1A is not expressed in connective tissue or bile duct lining cells. In the exposed group, hepatocytes in proximity to blood vessels displayed a stronger staining for CYP1A than hepatocytes more distant to blood circulation (Fig. 6A). This pattern was not observed in the same degree in the control group (Fig. 6B). No major differences were found between the different cell types in the expression of elongation factor (Fig. 7).

## Discussion

The effects of β-naphthoflavone (BNF), an aryl hydrocarbon ligand and CYP1A inducer, have been extensively studied in fishes [8-14]. In the current examination, the applied BNF dose increased the mRNA levels of CYP1A in liver by more than 100-fold. Using northern blot Grosvik *et al.*[10] showed that CYP1A mRNA increased eightfold in Atlantic salmon liver six hours after intraperitoneal injection of a similar BNF dose, with a maximum 48 hours after injection (14-fold). Their experiment showed that the induction (11-fold) was sustained throughout the eight-day experimental period. Other studies with BNF in fish have shown induction of CYP1A mRNA six hours after exposure followed by a decrease to near control levels 48 hours after injection in rainbow trout (*Oncorhynchus mykiss*), brook trout (*Salvelinus fontinalis*) and killifish (*Fundulus heteroclitus*) [8,15]. CYP1A induction was strong also in other organs of the exposed fish, especially in kidney tissue, which had very low baseline CYP1A mRNA levels. CYP1A is not constitutively expressed in many tissues in mammals or fish [16,17]. The results suggest that different cells and organs respond in a very distinct way to BNF exposure. Since the mechanisms behind CYP1A induction after BNF exposure are well known, no attempts will be made here to discuss these further. Seven days after intraperitonal injection of 50 mg BNF/kg body mass, the exposed fish showed highest CYP1A expression in the middle part (section 2 to 5) of the liver. Given that the average lifespan of eukaryotic mRNA rarely exceeds 24 hours [18], the data suggest that the liver receives a continuous supply of BNF via the blood from other organs that are able to induce CYP1A. Alternatively, accumulated BNF in the cytoplasm continues to re-induce CYP1A in the hepatocytes. In mouse (*Mus musculus*), Dalton *et al.*[19] have suggested that CYP1A mRNAs have a short half-life making it difficult to do reliable quantification. Our results indicate that CYP1A induction is strongest in the part of the liver closest to the portal vein, the large vein that carries blood from the stomach and intestine to the liver. This finding is also supported by the histological results, which indicate that the hepatocytes closest to the blood vessels show the strongest CYP1A induction. It was surprising to find that cells in the middle part of the liver had a higher CYP1A mRNA expression seven days after an acute exposure to BNF, given the homogenous nature of this tissue, which is comprised mainly of hepatocytes. The uneven mRNA expression of CYP1A throughout the liver further underlines how important it is to dissect and sample cells from the same part of the liver in future examinations of gene expression in bulk tissue specimens.

BNF exposure had much less effect on the liver mRNA levels of glutathione S-transferase (GST) compared to CYP1A. The transcriptional levels of this gene were two to four fold upregulated in the examined tissues. In exposed fish, this gene also shows an uneven expression throughout the liver, with the highest mRNA levels in sections 4 to 7. The GSTs are a family of phase II detoxifying enzymes that protect against injury from a number of endogenous and environmental chemicals. In mammals, the GST enzymes comprise a large supergene family. Their function has traditionally been considered to be the detoxification of electrophiles by glutathione conjugation during phase II biotransformation [20]. Donham *et al.*[21] characterized four GST classes from juvenile Chinook salmon (*Oncorhynchus tshawytscha*): π, μ, θ and α. Currently there is limited available information on orthologous GST genes in salmonids. The Atlantic salmon mRNA sequence used to design the PCR assay used in this study shows 96% similarity with sockeye salmon (*Oncorhynchus nerka*) π-class mRNA for GST (GeneBank BLASTX). Little is known about GST mRNA induction in fish exposed to various toxicants. Typically, only modest induction of overall GST activity has been reported under most conditions (2-fold or less) [22]. Trute *et al.*[23] recently characterized GSTs in Coho salmon (*Oncorhynchus kisutch*). They found two major GST isoforms in liver, the π and θ-class GSTs, but noted that they might have a limited capacity to conjugate substrates of various toxicants and endogenous compounds associated with cellular oxidative stress. The results presented here clearly show that the π class GST is inducible in Atlantic salmon, and indicate that the transcriptional levels are higher in hepatocytes in the middle parts of the liver in BNF-exposed fish, possible related to the supply of BNF from the blood.

In real-time quantitative reverse transcription PCR (qRT-PCR), data normalization is essential for accurate comparison of transcription measurements between different samples. In the current examination, a normalization factor was used to calculate mean normalized expression for the two target genes, based on the stability of three reference genes, i.e. β-actin, EF1A_B _and ARP. The use of reference genes represents the by far most common method for normalizing qRT-PCR data [24]. As there are no universal reference genes for all experimental systems, the choice of reference genes has to be carefully validated in each individual experiment [25]. The current results confirm that it is important to validate reference gene stability, as the most stable reference gene varied between the three studied tissues. The data also suggest that ARP seems to be downregulated in liver of fish exposed to BNF, and in general should not be used in calculation of the normalization factor in future toxicogenomic examinations in Atlantic salmon liver. In this study, however, ARP could be included as a reference gene in the liver examinations, since no differences were seen between the eight liver sections within the control- and exposed groups.

*In situ *hybridization (ISH) is a powerful technique that enables the detection of distinct mRNA species within individual cells in tissue sections, giving additional information about cell-specific transcription to support qRT-PCR data from bulk tissue specimens. CYP1A transcription was primarily found in the hepatocytes, with lower levels in other liver cell types. The data also suggest that CYP1A transcription in exposed fish was higher in cells in the vicinity of the blood vessels that penetrate the liver. Stegeman and Hahn [26] suggested that the vascular endothelium (lining of blood vessels) may be the primary target for the toxic action of CYP1A-inducing substances, since the endothelial cells are the first site of interaction with bloodborne toxicants. Based on the current ISH data in Atlantic salmon, the hepatocytes seem to be the major target for the toxic action of β-naphthoflavone, at least one week after a single-dose exposure. As expected, the reference gene EF1A_B _seems to be transcribed in all liver cell types. In order to obtain pure cell populations, a technique like laser-capture micro-dissection should be used. Even so, in most gene expression studies to date total RNA is normally extracted from bulk tissue samples; complex structures composed of heterogeneous mixtures of morphological and phenotypically distinct cell types. The histological results reported here suggest that gene expression analysis should be performed on as pure cell populations as possible. If bulk tissue samples are to be used, one should always check how evenly the target genes are expressed in every study.

## Conclusion

The current study shows that genes encoding phase I and phase II conjugating enzymes are unevenly transcribed in liver of Atlantic salmon acutely exposed to a single-dose of β-naphthoflavone. CYP1A transcription was higher in the middle section of the liver and in hepatocytes in the vicinity of blood vessels.

## Methods

### Fish treatment

About 130 seawater-adapted Atlantic salmon with an average weight of 350 g were obtained from the Matre Aquaculture Research Station in early April 2006, and maintained in a 1500 L tank with flow-through 34‰ seawater at the Institute of Marine Research, Bergen (60° 24' N), Norway. During the acclimation period, salmon were fed a commercial diet. A natural photoperiod was kept during the duration of the experiment. A total of 20 individuals were used in the experiment. All fish were starved for 24 hours before start of the experiment and during the experiment. The experiment was conducted between April 21st and 28^th ^2006. Seawater temperature was approximately 9°C during this period. On 21st of April, 20 individuals were randomly sampled and sedated by immersion in 50 mg/L metacaine (Norsk Medisinaldepot, Oslo, Norway). Sedated fish were given an intraperitoneal injection of β-naphthoflavone (BNF; Sigma Chemical Co., St. Louis, MO, USA; 50 mg/kg body mass) dissolved in soybean oil (10 mg/ml) as described by Grosvik *et al.*[10]. Each fish was injected with a 1 ml volume of BNF. Ten BNF-exposed fish were transferred into a 250 L outdoor tank where flow rate (10 L/min.) and temperature (9°C) were kept constant. The control, untreated fish were kept in their original 250 L tank with the same conditions during the entire experiment. Seven days after BNF exposure, 10 BNF-treated and 10 control salmon were anesthetized with metacaine and killed by a blow to the head before tissue sampling.

### Tissue sampling

Four tissues, liver, gills, head kidney and intestine, were sampled April 28^th ^2006 from a total of 20 Atlantic salmon with an average body mass of 381 ± 85 g and body length 33.9 ± 2.6 cm (fork length, mean ± S.D.). Average condition factor (c.f. = weight/length^-3^) was 0.96 ± 0.10 (mean ± S.D.). Tissue samples were dissected out as fast as possible, and immediately frozen in liquefied nitrogen. Each liver was cut into eight pieces for gene expression analysis, as shown in Fig. 1. Three specimens were sliced off for histology or *in situ *hybridization (ISH) examination, one from the proximal part of the organ, one from the mid part of the organ and one from the distal part of the organ. The mid section was cut in half, so that all together, four different sections were used for ISH from each liver. Tissue slices were fixed in 4% buffered paraformaldehyde (pH 7,6) overnight before dehydration and embedding in paraffin. The soft part of the gills was sliced off the gill arch. Data from the intestinal tissue study will be described in a separate work.

### RNA extraction

Tissues were thoroughly homogenized before RNA extraction with zirconium beads (4 mm) in a Retsch MM 301 homogenizer (Retsch GmbH, Haan, Germany). Total RNA was extracted using Trizol reagent (Invitrogen, Life Technologies, Carlsbad, CA, USA), according to the manufacturer's instructions and stored in 100 μl RNase-free MilliQ H_2_O. Genomic DNA was eliminated from the samples by DNase treatment according to the manufacturer's description (DNA-*free*, Ambion, Austin, TX, USA). The RNA was then stored at – 80°C before further processing.

### RNA quality and integrity

The quality of the RNA was assessed with the NanoDrop^® ^ND-1000 UV-Vis Spectrophotometer (NanoDrop Technologies, Wilmington, DE, USA) and the Agilent 2100 Bioanalyzer (Agilent Technologies, Palo Alto, CA, USA). 260/280 and 260/230 nm absorbance ratios of 1.8 – 2.0 indicates a pure RNA sample. The RNA 6000 Nano LabChip^® ^kit (Agilent Technologies, Palo Alto, CA, USA) was used to evaluate the integrity of the RNA. A 28S/18S rRNA ratio of 1.8–2.0 indicates undegraded RNA. The RNA integrity number (RIN) is a software tool developed by Agilent designed to help scientists estimate the integrity of total RNA samples. The software automatically assigns an integrity number to an eukaryote total RNA sample run on a Bioanalyzer RNA chip. A RIN of 10 indicates a pure RNA sample [27].

### Quantitative real-time RT-PCR

PCR primer sequences used for quantification of the genes encoding β-actin, elongation factor 1 alpha (EF1A_B_), acidic ribosomal protein (ARP), CYP1A and GST are shown in Table 1. PCR primers for β-actin were based on Atlantic salmon [Genbank: BG933897] and designed to span exon-exon borders of this gene, as deduced from corresponding genes in human and zebrafish. The EF1A_B _assay was based on the EST [Genbank: BG933853]. These two reference genes have also been used as references in real-time RT-PCR analyses of Atlantic salmon in other recent studies [28,29]. The mRNA sequence encoding ARP was obtained from GenBank accession numbers [Genbank: AY255630] (exon-exon borders were not considered). The ARP primer pair was obtained from an *Oncorhynchus tshawytscha *sequence. PCR primers for CYP1A and GST were obtained from Atlantic salmon accession numbers [Genbank: AF364076] and [Genbank: BQ036247], respectively. qPCR assays were designed using Primer Express 2.0 software (Applied Biosystems, Foster City, CA, USA) to select appropriate primer sequences from known salmonid genes. All assays produced single bands on a 2% agarose gel, verified with a One-Step RT-PCR kit from Qiagen (Qiagen, Chatsworth, CA, USA). The primer pairs amplified PCR products between 59–121 basepairs (bp) long, which is within the range of 50–150 bp suggested by Applied Biosystems for their TaqMan assays. However, in this study SYBR Green real-time PCR chemistry was applied, without the use of sequence-specific probes. RNA samples were subjected to DNase treatment to avoid genomic DNA contamination, since several of the primers did not span exon-exon borders. Amplified PCR products of all actual cDNAs were sequenced to ensure that the correct mRNA sequences were quantified. The fragments were sequenced with BigDye version 3.1 fluorescent chemistry (Applied Biosystems) and run on an ABI PRISM^® ^377 DNA sequencer at the University of Bergen Sequencing Facility.

**Table 1:** PCR primers and amplicon sizes for the five qPCR assays.

**Gene**	**Forward primer (5' to 3')**	**Reverse primer (5' to 3')**	**Product size (bp)**
β-actin	CCAAAGCCAACAGGGAGAA	AGGGACAACACTGCCTGGAT	92
EF1AB	TGCCCCTCCAGGATGTCTAC	CACGGCCCACAGGTACTG	59
ARP	TCATCCAATTGCTGGATGACTATC	CTTCCCACGCAAGGACAGA	101
CYP1A	TGGAGATCTTCCGGCACTCT	CAGGTGTCCTTGGGAATGGA	101
GST	ATTTTGGGACGGGCTGACA	CCTGGTGCTCTGCTCCAGTT	81

A two-step real-time RT-PCR protocol was developed to measure the mRNA levels of the genes in tissues of Atlantic salmon. The RT reactions were run in duplicate on 96-well reaction plates with the GeneAmp PCR 9700 machine (Applied Biosystems, Foster City, CA, USA) using TaqMan Reverse Transcription Reagent containing Multiscribe Reverse Transcriptase (50 U/μl), Applied Biosystems, Foster City, CA, USA). Twofold serial dilutions of total RNA were made for efficiency calculations. Six serial dilutions (1000 – 31 ng) in triplicates were analyzed by qRT-PCR in separate sample wells and the resulting Cts recorded. Input total RNA concentration was 500 ng in each reaction for all genes. Controls for no template (ntc) and controls for no amplification (nac) were run for each master mix, but not for every single sample. Reverse transcription was performed at 48°C for 60 min by using oligo dT primers (2.5 μM) for all genes in 30 μl total volume. The final concentration of the other chemicals in each RT reaction was: MgCl_2 _(5.5 mM), dNTP (500 mM of each), 10× TaqMan RT buffer (1×), RNase inhibitor (0.4 U/μl) and Multiscribe Reverse Transcriptase (1.67 U/μl).

2.5 μl cDNA for all genes from each RT reaction was transferred to a new 96-well reaction plate, and the real-time PCR run on the ABI Prism 7000 Sequence Detection System from AB. Real-time PCR was performed by using SYBR Green Master Mix (QuantiTect SYBR Green PCR kit, Qiagen, Chatsworth, CA, USA), which contains HotStar Taq DNA polymerase and gene specific primers (300 nM). PCR was achieved with a 15 min activation and denaturation step at 95°C, followed by 40 cycles of 15 s at 95°C and 60 s at 60°C. Baseline and threshold for Ct calculation were set automatically with the ABI Prism 7000 SDS software version 1.1, or set manually whenever necessary. The *geNorm *VBA applet for Microsoft Excel was used to determine a normalization factor from the three examined reference genes used to calculate mean normalized expression (MNE) for CYP1A and GST [25]. The Ct values were transformed to quantities using standard curves, according to the *geNorm *manual. *geNorm *determines the individual stability of a gene within a pool of genes, and calculates the stability according to the similarity of their expression profile by pair-wise comparison, using the geometric mean as a normalizing factor. The gene with the highest M, i.e. the least stable gene, is then excluded in a stepwise fashion until the most stable genes are determined. Here a normalizing factor based on all three examined reference genes was used to calculate the MNE.

### In situ hybridization (ISH)

Paraffin blocks were stored in RNase-free sealed boxes at 4°C until cutting (5 μm, Reichert 1330 Biocut microtome). Sections were mounted on Super Frost Plus slides (Bergman, Lillestrom, Norway) and dried overnight at 30°C. All slides were coded ensuring unknown identity until the examination was completed. To evaluate liver histology between treated and non-treated individuals, sections were stained with eosin (E). Figure 6 shows a representative example of hepatocyte morphology of a control liver and a treated liver.

Sections were dewaxed in xylene and hydrated to 50% ethanol before equilibration in phosphate buffered saline (PBS, pH 7.2). Sections were treated with 5 μg ml^-1 ^proteinase K for 20 min at 37°C, before 10 min post fixation in 4% PF – PBS. Prior to hybridization slides were deionised with acetic anhydride in 0.1 M triethanolamine, pH 8.0, for 10 min. Sections were incubated overnight in a humid chamber at 37°C in hybridization buffer (50% formamide, 10% dextran sulphate, 5× SSC (0.9 M NaCl, 0.09 M sodium citrate), 5× Denhardt's solution (0.1% (w/v) polyvinylpyrrolidone, 0.1% (w/v) and 60 μg/ml denatured salmon sperm DNA (250 μg/ml tRNA). The probes were added at 200 ng/ml.

Washes were done in 5× SSC with 30% formamide for 15 min, then twice in 0.2× SSC for 15 min at 54°C. Sections were blocked in 2% inactivated sheep serum and bovine serum in 0.1 M Tris saline pH 7.6. Detection of biotin conjugated cRNA probes was done by incubation with streptavidin alkaline phosphatase (AP) conjugate (Invitrogen, Carlsbad, CA, USA) overnight. Colour reaction was developed by incubating slides with nitroblue tetrazolium (NBT), 5-bromo, 4-chloro, 3-indolylphosphate (BCIP) (DakoCytomation Denmark A/S, Glostrup, Denmark) and indigenous AP blocked with 1 mM levamizole. Color development was stopped by washing slides in 0.01 M Tris, 0.01 M EDTA saline pH 8.0. Sections were counterstained in methyl green for 7 min prior to quick dehydration, cleared in xylene, and mounted in Entellan (Merck KGaA, Darmstadt, Germany). Sections were examined with an Olympus BX 51 light microscope (Olympus, Hamburg, Germany) and micrographs were obtained using an Olympus DP50 digital imaging system mounted on the microscope. All samples were always tested in triplicate on the same slide and on separate slides to enable the consideration of methodological intra- and intervariations. Omission of the specific probe in the hybridization reaction was used as an additional negative control.

The following biotinylated oligonucleotide probes were purchased, synthesized and labelled with 10 biotin molecules using 3' GreenStar™ technology (GenDetect.com Ltd., Auckland, New Zealand): EF1A_B _antisense (5'-GAC GAA GGG GCT TGT CTG TGG GGC GGG ATG GGG GCA GGA TGC TGT CCA-3') and CYP1A antisense (5'-GCG CTT GCC CAT GCC GAA TAC GAG CAC CTT CTC CCC CTC CAG CTT GTT-3'). The probes were specific and complementary to nucleotides 750–797 for EF1A_B _and nucleotides 1432–1479 for CYP1A and designed to avoid cross-reactivity towards the salmon genome, checked using the resources at The Salmon Genome Project [30]. Lyophilized probes were resuspended in RNase-free TE buffer (10 mM Tris, 1 mM EDTA, pH 8.0) at a concentration of 5 μg/500 μL and stored at -20°C.

### Statistics

The GraphPad Prism 4.0 software (GraphPad Software, Inc., San Diego, CA, USA) was used for the statistical analyses in this work. Nonparametric Mann-Whitney U-test was used to compare differences between the two groups of salmon examined. An alpha level of 0.05 was considered significant.

## Authors' contributions

PAO initiated the research and was responsible for all parts of the project, and also wrote the paper. KKL participated in planning, sampling and evaluation of the manuscript. ØS and MS did the *in situ *hybridization work and helped drafting those parts of the manuscript. All authors read and approved the manuscript.

## References

[B1] Ostrander GK (2000). The laboratory fish.

[B2] Alpini G, Phillips JO, Vroman B, LaRusso NF (1994). Recent advances in the isolation of liver cells. Hepatology.

[B3] Akiyoshi H, Inoue A (2004). Comparative histological study of teleost livers in relation to phylogeny. Zool Sci.

[B4] Buongiorno-Nardelli M, Amaldi F (1969). Autoradiographic detection of molecular hybrids between rRNA and DNA in tissue sections. Nature.

[B5] John HL, Birnstiel ML, Jones KW (1969). RNA-DNA hybrids at the cytological level. Nature.

[B6] Sanden M, Berntssen MH, Hemre GI (2006). Intracellular localization of dietary and naked DNA in intestinal tissue of Atlantic salmon, *Salmo Salar *L. using in situ hybridization. European Food Res Technol.

[B7] Qian X, Jin L, Lloyd RV (2004). In situ hybridization: Basic approaches and recent development. The J Histotechnol.

[B8] Kloepper-Sams P, Stegeman JJ (1989). The temporal relationships between P450E protein content, catalytic activity, and mRNA levels in the teleost *Fundulus heteroclitus *following treatment with B-naphthoflavone. Arch Biochem Biophys.

[B9] Goksoyr A, Andersson T, Buhler DR, Stegeman JJ, Williams DE, Forlin L (1991). Immunochemical cross-reactivity of beta-naphthoflavone-inducible cytochrome P450 (P4501A) in liver-microsomes from different fish species and rat. Fish Physiol Biochem.

[B10] Grosvik BE, Larsen HE, Goksoyr A (1997). Effects of piperonyl butoxide and beta-naphthoflavone on cytochrome P4501A expression and activity in Atlantic salmon (*Salmo salar *L). Environ Toxicol Chem.

[B11] Wilson JM, Vijayan MM, Kennedy CJ, Iwama GK, Moon TW (1998). Beta-naphthoflavone abolishes interrenal sensitivity to ACTH stimulation in rainbow trout. J Endocrinol.

[B12] Teles M, Gravato C, Pacheco M, Santos MA (2004). Juvenile sea bass biotransformation, genotoxic and endocrine responses to beta-naphthoflavone, 4-nonylphenol and 17 beta-estradiol individual and combined exposures. Chemosphere.

[B13] Teles M, Oliveira M, Pacheco M, Santos MA (2005). Endocrine and metabolic changes in *Anguilla anguilla *L. following exposure to beta-naphthoflavone – a microsomal enzyme inducer. Environ Int.

[B14] Chung-Davidson Y-W, Rees CB, Wu H, Yun S-S, Li W (2004). Beta-naphthoflavone induction of CYP1A in brain of juvenile lake trout (*Salvelinus namaycush *Walbaum). J Exp Biol.

[B15] Haasch ML, Wejksnora PJ, Stegeman JJ, Lech JJ (1989). Cloned rainbow trout liver P450 complementary DNA as a potential environmental monitor. Toxicol Appl Pharmacol.

[B16] Campbell SJ, Henderson CJ, Anthony DC, Davidson D, Clark AJ, Wolf CR (2005). The murine CYP1A1 gene is expressed in a restricted spatial and temporal pattern during embryonic development. J Biol Chem.

[B17] Zodrow JM, Stegeman JJ, Tanguay RL (2004). Histological analysis of acute toxicity of 2,3,7,8-tetrachlorodibenzo-p-dioxin (TCDD) in zebrafish. Aq Toxicol.

[B18] Meyer S, Temme C, Wahle E (2004). Messenger RNA turnover in eukaryotes: pathways and enzymes. Critical Rev Biochem Mol Biol.

[B19] Dalton TP, Dieter MZ, Matlib RS, Childs NL, Shertzer HG, Genter MB, Nebert DW (2000). Targeted knockout of CYP1A1 gene does not alter hepatic constitutive expression of other genes in the mouse [Ah] battery. Biochem Biophys Res Comm.

[B20] Strange RC, Spiteri MA, Ramachandran S, Fryer AA (2001). Glutathione-S-transferase family of enzymes. Mutation Res.

[B21] Donham RT, Morin D, Jewell WT, Lame MW, Segall HJ, Tjeerdema RS (2005). Characterization of cytosolic glutathione S-transferases in juvenile Chinook salmon (*Oncorhynchus tshawytscha*). Aq Toxicol.

[B22] Henson KL, Stauffer G, Gallagher EP (2001). Induction of glutathione S-transferase activity and protein expression in brown bullhead (*Ameiurus nebulosus*) liver by ethoxyquin. Toxicol Sci.

[B23] Trute M, Gallis B, Doneanu C, Shaffer S, Goodlett D, Gallagher E (2007). Characterization of hepatic glutathione S-transferases in coho salmon (*Oncorhynchus kisutch*). Aq Toxicol.

[B24] Huggett J, Dheda K, Bustin S, Dorak MT (2006). Normalization. Real-time PCR.

[B25] Vandesompele J, Preter KD, Pattyn F, Poppe B, Roy NV, Paepe AD, Speleman F (2002). Accurate normalization of real-time quantitative RT-PCR data by geometric averaging of multiple internal control genes. Genome Biol.

[B26] Stegeman JJ, Hahn ME, Malins DC, Ostrander GK (1993). Biochemistry and molecular biology of monooxygenases: Current perspectives on forms, functions, and regulation of cytochrome P450 in aquatic species. Aquatic Toxicology: Molecular, Biochemical, and Cellular Perspectives.

[B27] Schroeder A, Mueller O, Stocker S, Salowsky R, Leiber M, Gassmann M, Lightfoot S, Menzel W, Granzow M, Ragg T (2006). The RIN: an RNA integrity number for assigning integrity values to RNA measurements. BMC Mol Biol.

[B28] Moore LJ, Somamoto T, Lie KK, Dijkstra JM, Hordvik I (2005). Characterisation of salmon and trout CD8α and CD8β. Mol Immunol.

[B29] Olsvik PA, Lie KK, Jordal AE, Nilsen TO, Hordvik I (2005). Evaluation of potential reference genes in real time RT-PCR studies of Atlantic salmon. BMC Mol Biol.

[B30] The Salmon Genome Project. http://www.salmongenome.no/cgi-bin/sgp.cgi.

